# Working at the Grassroots Level: A Qualitative Exploration of Frontline Health Workers’ Experiences in Tribal Maternal and Reproductive Health Services

**DOI:** 10.7759/cureus.106932

**Published:** 2026-04-13

**Authors:** Manu Krishnan, Kimneihat Vaiphei, Pracheth Raghuveer

**Affiliations:** 1 Department of Psychiatric Social Work, National Institute of Mental Health and Neuro Sciences, Bengaluru, IND; 2 Department of Epidemiology, National Institute of Mental Health and Neuro Sciences, Bengaluru, IND

**Keywords:** anganwadi, asha (accredited social health activists), community health workers, cultural competence, grassroots healthcare, indigenous maternal health, low-resource settings, marginalized community, non-governmental organization (ngo), reproductive health services

## Abstract

Background

Frontline health workers play a crucial role in bridging gaps between health systems and marginalized communities. However, there is limited evidence of their experiences within tribal maternal and reproductive health services. This study aimed to explore the nature of work, lived experiences, core competencies, challenges, and motivational factors of frontline health workers providing maternal and reproductive health services in a tribal setting in Kerala.

Methods

A qualitative exploratory study using a narrative inquiry approach was conducted in the Attapadi tribal settlement of Palakkad district, Kerala. Eighteen frontline health workers, including Accredited Social Health Activist (ASHA) workers, Anganwadi workers, and non-governmental organization (NGO) field staff with a minimum of five years of experience, were purposively selected. In-depth semi-structured interviews were conducted in Malayalam. Data were transcribed verbatim and analyzed manually using thematic analysis.

Results

Frontline health workers performed multifaceted roles extending beyond formal responsibilities, including home-based service delivery, health education, follow-up care, and facilitation of government welfare schemes. Cultural competence, trust-building, context-specific communication, and problem-solving in low-resource settings were identified as core competencies. Major challenges included difficult terrain, transportation barriers, cultural resistance, staff shortages, heavy workload, and emotional stress. Despite these constraints, strong commitment to community welfare, personal satisfaction from positive maternal outcomes, social recognition, and peer support sustained workers’ motivation and resilience.

Conclusion

Strengthening policy support, enhancing culturally sensitive training, addressing workload and emotional well-being, and improving health infrastructure are essential to sustain their contributions and advance equitable maternal health outcomes among tribal populations.

## Introduction

Reproductive health is a fundamental human right for all women. Its implications extend beyond individual women and their families, impacting broader outcomes such as economic stability, productivity, and the long-term social, economic, and developmental progress of a nation. However, women and adolescents in low- and middle-income countries continue to face significant health challenges [1]. Maternal and reproductive health care during the perinatal and postpartum period has traditionally emphasized the prevention of infant and maternal mortality and morbidity [2]. Utilization of maternal health services plays a crucial role in reducing maternal deaths and enhancing women’s overall reproductive health outcomes [3,4]. However, limited availability of maternal health services and underutilization of existing available services are commonly observed among populations with the greatest need, particularly marginalized and socially disadvantaged groups, including tribal and low socioeconomic status (SES) populations [2,5].

In India, remarkable disparities are present in the utilization of maternal health services, including antenatal and maternity care, across states and among different population groups within states. These inequalities are particularly pronounced among populations stratified by socioeconomic disadvantage communities [6]. Such disparities reflect persistent maternal health inequities in India. Health inequities refer to differences in access to health services or health outcomes that are considered avoidable, unjust, and unfair [7].

Compared with the emphasis placed on improving the quality of and access to health services, the influence of women’s socio-economic conditions on maternal health has received relatively limited attention and care [6]. There is growing concern that many health intervention programs remain predominantly supply-oriented, often overlooking the social factors that shape demand for care, access to services, and their effective utilization [8]. Evidence suggests that women’s educational and social status, household economic conditions, and decision-making power are strongly interconnected with maternal health-seeking behaviors [9].

To address these challenges, frontline health workers represent a critical and readily available resource in low- and middle-income countries. They play a pivotal role in bridging gaps in the utilization of maternal health and nutrition services and in reducing maternal mortality at the community level [10]. In India, auxiliary nurse midwives (ANMs), accredited social health activists (ASHAs), and Anganwadi workers (AWWs) form an integral part of the public health system and constitute key human resources for the delivery of maternal and neonatal health services [11]. These workers provide health-related information and engage with women and their families to enhance the uptake of maternal and antenatal healthcare services, while also supervising and monitoring the implementation of national and state-wise health programs within communities [10], including marginalised and vulnerable populations.

This study seeks to explore the nature of work undertaken by frontline health workers, their personal experiences, challenges, and opportunities, as well as their suggestions for improving the implementation of maternal and reproductive health services in limited resource settings, particularly within tribal areas.

## Materials and methods

Study design

This study employed a qualitative exploratory research design using a narrative inquiry approach to capture in-depth information from participants. The narrative approach facilitated the collection of experience-based accounts, enabling participants to share their stories [12,13] and lived experiences related to their work in maternal and reproductive health services. Such methods have become essential for generating deep insights and developing a nuanced understanding of the experiences [14,15] of field health staff engaged in maternal and reproductive health care within marginalized settings.

Study setting

According to the Census of India, 2011, the Scheduled Tribes population in Kerala constitutes 484,839 individuals, accounting for 1.45% of the entire state’s population. There are 36 tribal communities, including five particularly vulnerable tribal groups. Tribal populations are predominantly settled in the Western Ghats region, with the highest proportion living in Wayanad district, followed by Idukki and Palakkad [16]. The present study was conducted in the Attapadi tribal settlement. Within the Palakkad district, Attapadi represents a major planned tribal settlement. Located in the Nilgiri hills in the Western Ghats. Attapadi spans approximately 750 km^2^ and serves as a buffer zone to the Silent Valley National Park. The settlement comprises three panchayats (local administration) named Sholayur, Pudhur, and Agali. The settlement is home to three tribal communities: Mudugars, Irulars, and Kurumbars [17].

The major rationale for selecting the Attapadi settlement in particular is that it represents one of the regions in Kerala with a persistently high infant mortality rate. While Kerala has achieved notable success in reducing infant mortality to below the national average, Attapadi remains an exception to this trend. This disparity prompted the researcher to focus on Attapadi to gather in-depth data from frontline health workers functioning at the grassroots level.

Participants and data collection

Participants were selected using a purposive sampling technique. A total of 18 participants were included in the study. The sample comprised grassroots-level field health workers from both government and non-governmental organizations. Participants included ASHAs, AWWs, and field staff from various non-governmental organizations (NGOs). All participants had a minimum of five years of experience in providing maternal and reproductive health services within the study area.

Data were collected between February 2025 and October 2025. Confidentiality and anonymity were strictly maintained throughout the data collection process. Interviews were conducted in Malayalam, the language preferred by the participants. Each interview lasted approximately 60-90 minutes and was conducted either at the participants’ homes or at their workplaces, ensuring privacy and confidentiality. Every interview started with a formal introduction and an ice-breaker question to establish rapport, followed by the collection of socio-demographic data. In-depth qualitative interviews were then conducted using a semi-structured interview guide (see Appendices), which was validated by a group of professionals, including psychiatrists, epidemiologists, social workers, and other community health professionals. However, it was refined iteratively during the initial phase of data collection. Based on insights from early interviews and emerging themes, certain questions were rephrased, additional probes were incorporated, and the sequence of questions was adjusted to improve clarity and depth of responses.

Participants’ queries were addressed appropriately based on the researcher’s knowledge. All interviews were audio-recorded, and detailed field notes were maintained to document interview duration, participant behaviors, emotional expressions, non-verbal cues, and contextual observations. 

Description of Participants

ASHA: ASHAs are community-based workers under the National Rural Health Mission, usually from the same local community [18]. Their work is largely individual and field-based, involving home visits, pregnancy tracking, and linking families to health services. Due to dispersed hamlets and difficult terrain, they often work independently, leading to increased travel and workload.

Anganwadi Workers: AWWs operate under the Integrated Child Development Services (ICDS) and are primarily centre-based, focusing on nutrition, child development, and maternal support [19,20]. However, in this setting, they also conducted frequent field visits, combining centre-based and outreach work, which added to their responsibilities.

Project workers: Project workers, part of the Community Maternity Care Project, function in team-based structures, including nurses and social workers [21]. Their work involves coordinated home visits, monitoring, and follow-up of high-risk pregnancies. This team-based approach allows shared responsibilities and better workload distribution.

Ethical considerations

This study was approved by the Institutional Ethics Committee of the National Institute of Mental Health and Neuro Sciences (NIMHANS), Bengaluru (approval number: 077-2559). The study was also reviewed and prospectively registered in the Clinical Trials Registry of India (CTRI) (registration number: CTRI/2025/03/082016). Subsequently, formal permissions were obtained from the Scheduled Tribes Development Department (STDD/4549/2024-B2) and the Kerala Forest Department (KFDHQ/2451/2025-CWW-WL10) before initiating data collection in the tribal settlements.

Written informed consent was obtained from all literate participants. For participants with limited formal education, witnessed verbal consent was secured after providing a clear explanation of the study’s purpose, methods, and voluntary nature. Throughout the study, ethical principles of autonomy, confidentiality, and anonymity were strictly upheld to ensure participants’ rights and dignity were protected.

Data analysis

**Figure 1:**
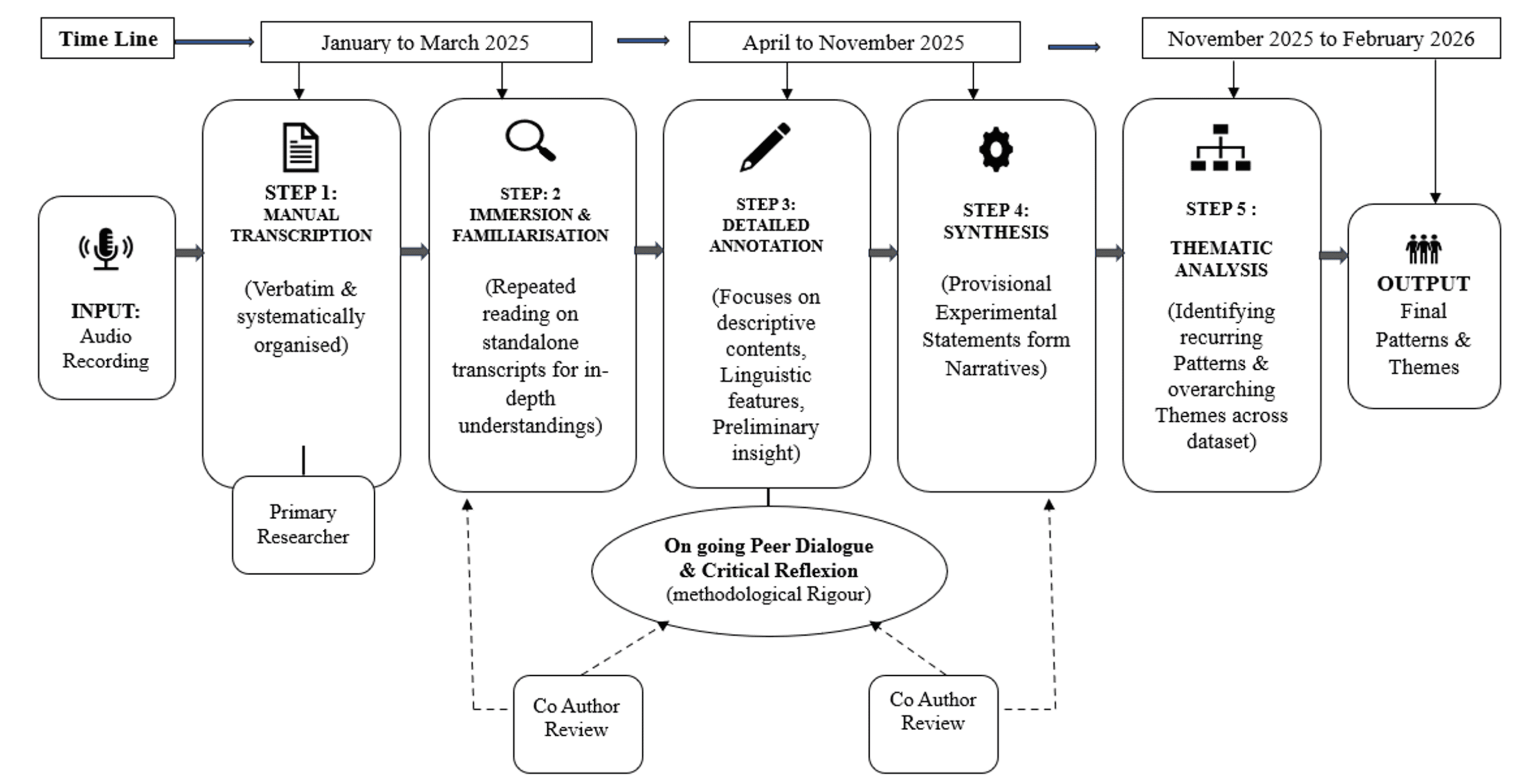
The qualitative data analysis process Image created by authors using Microsoft Word (Microsoft 365, Version 2603), Microsoft Corporation, Redmond, Washington, United States

Data analysis was conducted manually. Figure 1 illustrates the manual process followed for qualitative data analysis in this study. Audio recordings were transcribed manually as the initial step of the analysis, and all interviews were transcribed verbatim and systematically organized. The primary researcher led the analytic process, while co-authors independently reviewed analytic decisions at different stages to enhance methodological rigor through ongoing peer dialogue and critical reflection. Each transcript was treated as a standalone account. The researcher read each transcript multiple times to gain an in-depth understanding of the individual experiences of frontline health workers. Detailed annotations were made focusing on descriptive content, linguistic features, and preliminary interpretative insights [22]. These were subsequently synthesized into provisional experiential statements capturing key aspects of each participant’s narrative. The data were then subjected to thematic analysis to identify recurring patterns and overarching themes across the dataset [23]. A preliminary codebook was developed by the primary researcher and refined during the analysis. Peer review among co-authors was conducted to cross-check interpretations and ensure consistency in coding and theme development. Although qualitative software was not used and formal inter-coder reliability statistics were not calculated, multiple rounds of discussion and consensus among the researchers were undertaken to strengthen the dependability and transparency of the analysis.

Study trustworthiness and reflexivity

**Figure 2:**
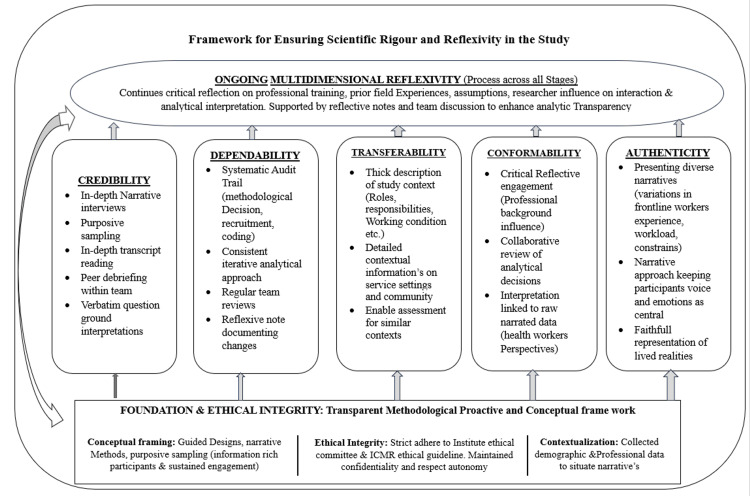
Framework for ensuring scientific rigor and reflexivity in the study ICMR: Indian Council of Medical Research Image created by authors using Microsoft Word (Microsoft 365, Version 2603), Microsoft Corporation, Redmond, Washington, United States

Scientific rigour or trustworthiness was systematically tested across all stages of the research process through transparent and rigorous methodological practices, as shown in Figure 2. A clearly articulated and systematic conceptual framework guided the selection of the research designs, narrative methods, and informed recruitment and sampling strategies aimed at including information-rich participants with sustained engagement in grassroots health work. Ethical integrity was ensured through strict adherence to informed consent, confidentiality, and respect for participants’ autonomy. Demographic and professional contextual information was collected to situate participants’ narratives within their service settings, thereby strengthening credibility and interpretive depth.

Reflexivity was embedded as an ongoing and multidimensional process throughout the research, data collection, coding, and analysis. The research team continuously reflected on how their professional training, prior field experience, engagement with community and health systems, and prior understanding about the study population might influence researcher interaction with participants and analytic interpretations, coding, and theme generation. This was overcome with reflective notes and continuous discussion within the research team. Reflexive discussions among team members supported critical analysis of assumptions and enhanced analytic transparency, ensuring that participants’ voices remained central to the data analysis.

## Results

A total of 18 participants were interviewed for the study. The socio-demographic details of the participants are presented in Table 1.

**Table 1:** Sociodemographic profile of the study participants (N=16) ASHA: Accredited Social Health Activist

Characteristics	Categories	Frequency (Percentage)
Age groups (Mean age = 32.8)	20-30 years	8 (44.4 %)
	31-40 years	6 (33.3 %)
	41-50 years	4 (22.2%)
Education	Higher secondary	10 (55.5%)
	Graduation	2 (11.1 %)
	General Nursing and Midwifery	4 (22.2 %)
	Auxiliary Nurse Midwife	2 (11.1 %)
Employment Status	Government	12 (66.6 %)
	Non-government Organisations	6 (33.3 %)
Nature of Work	ASHA Workers	6 (33.3 %)
	Anganwadi Workers	6 (33.3 %)
	Project Workers	6 (33.3 %)
Years of Experience in Health-related work	5 years	6 (33.3%)
	6 to 10 years	4 (22.2 %)
	More than 10 years	8 (44.4 %)

A total of four major themes and 19 subthemes emerged from the analysis, derived from 287 initial codes and supported by 193 participant quotations. The thematic analysis of data obtained from participant interviews conducted in this study is given below.

Theme 1: nature of grassroots maternal and reproductive health work

Frontline health workers performed multiple tasks to ensure the delivery of maternal and reproductive health services at the grassroots level. Although their roles and responsibilities were formally defined, they often took on additional tasks beyond their assigned duties to address emerging needs. Their work followed a holistic approach to healthcare extending beyond women of reproductive age to include husbands and other family members. This inclusive approach reflects the realities of service delivery in lower socioeconomic and tribal settings where family and community involvement play a major role in maternal and reproductive health outcomes. Anganwadi centres functioned as key service points for nutrition support, growth monitoring, and health education sessions for women and children. However, due to low institutional access and geographical barriers, a significant portion of care was provided through home-based visits across dispersed tribal hamlets (oorus). In addition, community health camps and awareness programmes were periodically organized in remote areas to improve access to antenatal check-ups and basic diagnostic services. These camps and programmes were conducted in public spaces such as panchayat halls, temple halls, and Anganwadi centres, depending on local availability.

Home Visits and Community-Based Service Delivery

Frontline health workers regularly conducted home visits to reach pregnant women and mothers who may not have access to health facilities. These visits allowed them to provide services directly within the community, especially in remote and tribal areas where transportation, resources, and access to hospitals are limited. Frontline health workers maintained a strong rapport with community members. As a result, when a woman became pregnant, the information was often first shared with ASHA workers or Anganwadi staff. This information is then reported to the ICDS system to ensure early registration and follow-up. During home visits, these workers provided pregnancy-related counselling to women and their family members. Their focus included maternal health, nutrition, pregnancy-related care, maternal mental health, and strengthening family support systems. In addition, ASHA and AWWs assisted women in accessing various government welfare schemes and benefits available for pregnant and lactating mothers.

“Every month, we visit their homes and conduct counselling for pregnant women and their spouses. They are very reluctant to come to the hospital due to various issues, so we go there and do the work” ~ Project Staff 6

“Some of them come for ANC visits. We collect samples from their homes. Mostly, we check blood pressure and collect blood and urine samples. Later, we inform them about the results. This does not cost anything for them, so they are happy to cooperate with us” ~ Project Staff 4.

Maternal and Reproductive Health Education

Health education and information facilitation were core responsibilities of frontline health workers involving maternal and reproductive health. They provided information and knowledge on antenatal care, institutional delivery, nutrition, family planning, infant development, postnatal care, and, in some instances, maternal mental health using local language and culturally appropriate methods. They used simple non-technical language while communicating with women and their family members, which helped enhance understanding and acceptance of health-related information. They also provided informational pamphlets during home visits and regularly cross-checked antenatal cards to monitor service utilisation and ensure continuity of care.

“We have to educate not only the mother, but mostly the mothers-in-law and husbands. They are only available in their homes. Mostly, we discuss pregnancy, trimesters, ANC visits, food, and nutrition. In some cases, if they are flagged as high risk, we also discuss emergency situations” ~ Project Staff 5

In addition to these responsibilities, frontline health workers also conducted contraceptive counselling and family planning sessions for women and their families, further expanding their role in reproductive health decision-making at the community level.

“We also discuss contraception plans with them. Sometimes it is very difficult to make them aware about it. We are encouraging them to use Copper T, which is freely available in our hospital” ~ ASHA 5

Follow-up Care and Referral Coordination

Frontline workers played a crucial role in follow-up care by monitoring high-risk pregnancies, immunisation schedules, and postnatal recovery. They also coordinated referrals to primary health centres or hospitals and often accompanied women to ensure timely and continuous care. In some instances, they served as the first point of contact for the indigenous population. Regular home visits enabled them to identify high-risk pregnancies at an early stage and mark these cases as priority or “red flag” cases within their records.

“We provide our number to the family members of women who are at high risk. So they can call us at any time, and we can arrange an ambulance and inform the concerned hospital before they reach” ~ ASHA 4

Women identified as high risk received increased attention and monitoring, closer follow-up, and timely referrals from the service providers. This practice helped reduce potential complications and minimize adverse effects on the mother and infant.

Work Beyond Fixed Working Hours

The work timing and usual schedules frequently extended fixed duty hours. Emergencies and community needs often required them to be available at night or during holidays, highlighting the flexible and demanding nature of grassroots health work. Community members often approached ASHA and AWWs for immediate assistance. Due to limited resources and the absence of alternative support options, these workers became the primary point of contact. This situation created uncertainty in working hours, as they were frequently required to respond to community needs beyond scheduled duty times.

“hm…… (Smiling) Basically, we don’t have a specific work routine. We plan our work every day. Sometimes the work ends before 3, but mostly it goes till 6 or 7” ~ ASHA 1

Mediators of Government Assistance

Frontline health workers acted as key facilitators in linking eligible pregnant women to government assistance schemes. One of the important schemes supported through their work was the Pradhan Mantri Matru Vandana Yojana (PMMVY), a maternity benefit programme implemented under the Ministry of Women and Child Development as part of the Mission Shakti scheme [24,25]. The scheme provided monetary support of ₹5,000 for the first child and ₹6,000 for the second girl child under PMMVY 2.0. ASHA and AWWs collected the required beneficiary information and forwarded applications to the ICDS supervisors for processing. In addition to financial assistance, nutritional food supplements were provided through Anganwadi centres, and free meals were facilitated for pregnant and lactating mothers. These frontline workers played a central role in ensuring awareness, enrolment, follow-up, and utilisation of these schemes.

“Central government schemes are there. Women will get 5,000 rupees. It is given as instalments. For that, we have to report to the ICDS supervisor and collect Aadhaar, medical reports, and passbook. They will process that” ~ AWW 4

In addition, ASHA and AWWs provided support for infant immunisation by conducting awareness activities, making arrangements for vaccination sessions, and assisting in the organisation of immunisation and polio vaccination camps. Alongside maternal and child health services, they actively participated in community-based pain and palliative care activities, as well as in pandemic response and epidemic control programmes.

Theme 2: core competencies for working at the grassroots level

The community-based holistic approach adopted by frontline health workers is highly impactful in grassroots health care and service provision. They applied key competencies and practical strategies in their daily practices to engage with the family and community. Years of experience and in-depth knowledge of local culture and community practices helped them develop these competencies and strengthen community engagement. Indigenous populations often strongly adhere to their traditional values and belief systems, and modern medical language or approaches were not always accepted easily. The experiential knowledge and culturally and locally sensitive working methods of frontline workers were thus highly valuable in ensuring effective maternal and reproductive health service delivery with the indigenous community.

Most frontline health workers belonged to the same region and came from different tribal communities; therefore, they were familiar with the common beliefs, practices, and social norms of the region.

Cultural Understanding of Tribal Communities

Frontline workers possessed a strong understanding of tribal culture, including customs, beliefs, and traditional practices related to pregnancy and childbirth. This cultural familiarity helped them deliver health services in a respectful manner and reduced resistance to maternal and reproductive health interventions of the government. Religious and traditional practices could sometimes increase health risks for women. For example, a frontline health worker in a tribal hamlet encountered a pregnant woman identified as high-risk due to a history of previous abortions, who repeatedly missed antenatal check-ups. The family believed her condition was caused by “dheiva kopam” (divine displeasure) and prioritized performing *pooja* (worship) and *vazhipadu* (traditional offerings) at a local temple rather than seeking medical care. Despite repeated encouragement from the health worker, the family insisted on completing religious rituals before considering hospital visits. Also, during festivals such as Maha Shivaratri, Vishu, and Onam, they refused to attend scheduled appointments and hospital visits. However, strong opposition to these practices could damage the rapport between the health system and the community. Therefore, the frontline health worker negotiated health interventions within the cultural context of the community. This approach, followed by the health workers in the region, requires a strong understanding of cultural backgrounds, beliefs, and practices to ensure acceptance while promoting safer maternal and reproductive health behaviours.

“I belong to the Irula community, so I know the culture and language. They trust me more than MBBS or MD doctors.” (smiling) ~ ASHA 5

“Use simple and accepted dressing style (Figure 1, 2). Greet and smile at them wherever we see them. Use polite and respectful language; they will accept you also” ~ Project Staff 1

Context-Specific Communication Skills

These frontline workers communicated health information using local languages and culturally appropriate expressions. By avoiding technical terms and using simple explanations, they ensured that women and family members clearly understood health advice and recommended practices.

The Attappady tribal communities primarily spoke their own indigenous languages. These languages were primarily a blend of Malayalam and Tamil, with noticeable influences from Kannada and Telugu as well. Among the communities, the Irula people commonly spoke the Irula language, the Kurumba community used the Betta Kurumba language, and the Muduga community spoke its own distinct dialect [26]. Each of these languages and dialects shows the unique cultural and custom identity of the tribal groups while also showing linguistic interactions with neighbouring regions. Providing health information in the same language spoken by the community enhanced acceptance and trust at the community level and facilitated more effective engagement by frontline health workers.

“True. I am not using any medical language. I always try to explain everything in our language; otherwise, they won’t understand" ~ Project Staff 3

Trust-Building with Community Members

Establishing rapport was crucial when working with Indigenous populations, although it could sometimes be challenging. However, once trust was built, relationships often became strong, enduring, and deeply respectful. Often, home visits and long-term engagement enabled health workers to build trust within the community. This trust helped encourage women and families to seek timely support and share sensitive health information and medical advice. Wearing common dressing styles, using the local language, respecting tribal traditions, maintaining a polite tone during conversations, and showing respect to elder community leaders, even though they were critical of or opposed to modern treatment systems, were effective ways to build rapport with Indigenous communities.

“I am very happy to conduct home visits. I like their gestures and the way they welcome us into their homes. What I observe is that if you respect them, they will respect you also.” ~ ASHA 6

“Yes, my people are very sensitive. If you do not hurt our emotions and beliefs, they will accept you.” ~ AWW 1

Problem Solving in Low-Resource Settings

Geographical isolation and limited resources remained major challenges in the area. To address these constraints, frontline health workers adopted problem-solving approaches in their daily practice. They carefully planned their daily tasks and schedules based on local needs and contextual limitations. In some instances, they took the initiative to organize health camps in remote interior areas, ensuring medical check-ups and basic health services for women. The workers also actively supported and assisted research initiatives aimed at improving health-related knowledge within the community. Another important strategy involved collaboration with women’s self-help groups, particularly Kudumbashree units. Almost every ooru (hamlet) had two to three Kudumbashree groups, which enabled health workers to conduct awareness sessions during regular meetings [27]. This approach allowed health information to reach pregnant women and their families multiple times and from multiple trusted sources. Such context-specific, problem-solving strategies were particularly effective in overcoming challenges and strengthening service delivery in resource-limited settings.

“My approach is to involve others also in the work. I also take help from Kudumbashree workers to get information about pregnant women.” ~ AWW 3

Early Identification of Maternal and Reproductive Health Risks

Through continuous monitoring and repeated interactions, health workers were able to identify early signs of high-risk pregnancies and reproductive health concerns. This early detection facilitated timely referrals and follow-ups, which were crucial for preventing complications and improving maternal health outcomes. In the settlement specifically, a significant number of underweight cases were identified. In response, 10 health workers were mobilized to identify affected individuals and facilitate the distribution of necessary food items and nutritional support. Additionally, the community had reported a prevalence of other serious health concerns, including anemia, spontaneous abortions, and miscarriages. Therefore, early identification of pregnancy and timely interventions for both high and low-risk cases helped reduce adverse outcomes.

Theme 3: challenges faced in service delivery

Even though frontline health workers were central figures in improving community health and social well-being, they continued to face various challenges and problems in their daily work. The sub-themes that emerged are reported below.

Difficult Terrain and Transportation Barriers

The geographically isolated tribal areas were marked by hilly terrain, forest paths, poorly maintained roads, and poor transportation facilities. Seasonal challenges, especially during monsoons and scorching summer, often made villages inaccessible, delaying antenatal and home visits, and emergency referrals. Most frontline health workers relied on motorbikes as their primary mode of transportation to reach remote tribal villages. However, seasonal changes often made these routes unsafe or impassable, significantly affecting their ability to deliver timely maternal and reproductive health services to the indigenous population.

“*It is hard to reach some oorus. We have data on pregnant women. In one ooru, maybe only one or two women are there, but we have to travel around 8 to 10 kilometres to reach one home from another home. It is difficult. I ride my bike, but during the monsoon all the roads become impassable. During the summer, the temperature is very high and it causes dehydration.” ~ AHSA 2*

Another major challenge reported by participants was that most ooru were surrounded by dense forest areas where wildlife activity was common even during early morning hours. Travelling through these routes was often perceived as unsafe due to the risk of animal encounters. Health workers reported frequent sightings of elephants, wild boars, and leopards, as well as the presence of poisonous snakes such as vipers, Indian spectacled cobras, etc. These conditions significantly restricted mobility, increased travel-related anxiety and fear.

“*Yes sir… elephants and wild boars are common here. You can see them even now.” ~ AWW 5*

Limited Health Infrastructure and Resources

Compared to many other tribal regions, Attappady had relatively better-maintained government hospitals and treatment facilities, which reflected sustained efforts by the government and NGOs. However, several challenges continued to persist. Participants pointed out gaps in advanced infrastructure, periodic shortages of essential medicines, and limited availability of diagnostic services. Frontline health workers often had to manage maternal and reproductive health needs with minimal resources, frequently relying on their own initiative to bridge service gaps. While government and NGO initiatives were widely appreciated, participants noted that late-night emergencies were often referred to hospitals in nearby towns due to limited facilities, which increased travel time and posed additional risks to mothers and newborns. The frontline health workers, including ASHAs and AWWs, resided within the same tribal communities they served and typically lived in their own homes. This close proximity enabled continuous engagement, better trust-building, and quicker response to community needs. In contrast, other healthcare professionals, such as doctors, nurses, and paramedical staff, were generally posted from outside the region. These personnel often depended on rented accommodation within or near the study area for their stay.

Resistance Influenced by Traditional Beliefs

Health workers frequently encountered strong resistance rooted in long-standing cultural beliefs and traditional practices related to pregnancy and childbirth. Repeated home visits were often necessary to gradually change the perspectives of pregnant women and their families. Deeply rooted myths and misconceptions posed significant barriers to maternal care. For instance, some women concealed their pregnancies due to fears that others might cast the “Dhristhri” (evil eye) or practice “Koodothram” black magic out of jealousy, which they believed could harm the unborn child. Maternal mental health concerns were also frequently interpreted as spirit possession or demonic attacks, sometimes linked to lunar phases such as full moon or new moon periods. These beliefs often delayed disclosure, care-seeking, and acceptance of medical interventions, making sustained engagement and trust building in frontline health work.

“Yes, of course. We are so dependent on our cultural belief systems. Sometimes it becomes very hard to change these narratives. Especially certain beliefs. I don’t even know if I can call them myths but anyway, they are very difficult to change.” ~ AWW 6

Workload Pressure and Staff Shortages

Due to persistent trained resources in tribal health settings, frontline workers were responsible for covering multiple villages and performing overlapping duties. This increased workload affected their ability to provide sustained individualised care to pregnant and lactating women. Participants noted that although the number of households assigned to them was sometimes relatively small, the geographical area they were required to cover was extensive. Each ooru (hamlet) was often located 10-25 kilometres apart, separated by dense forest terrain. This increases their workload.

“During this time also, I have to finish a lot of work, especially reporting. Within this month, by the 28th, I have to upload all the pregnant women and infant-feeding mothers’ reports to the block. I think we really need more trained people to manage these tasks. You know, during the COVID time we struggled a lot. We were assigned COVID duties too.” ~ AWW 2

In addition to maternal and reproductive health responsibilities, ASHA and AWWs were assigned multiple other duties, positioning them as central figures in promoting overall community well-being. Participants described being involved in a wide range of activities, including health promotion, poverty alleviation initiatives, early childhood care and education, adolescent health programmes, birth and death census and reporting, pain and palliative care services, and geriatric care. Their roles also extended to routine data collection, annual record maintenance, coordination with other healthcare professionals, and conducting regular home visits. The wide range of responsibilities assigned to ASHA and AWWs substantially increased their workload.

Emotional Stress and Fatigue

The wide range of duties and tasks, persistent staff shortages, systemic challenges, inadequate salaries and benefits, and strong cultural resistance collectively contributed to emotional difficulties among frontline health workers. Participants reported experiencing frequent fatigue, high levels of stress, and emotional exhaustion. Many workers noted that they had not received adequate training to manage grief, distress, or negative emotional expressions from mothers and families, which further affected their mental well-being. Over time, this emotional burden led to feelings of deep sadness and depressive symptoms among some workers. Disturbed sleep patterns, reduced sleep duration, and poor sleep quality were also commonly reported, largely due to increased workload and work-related stress. As most frontline healthcare workers were women, balancing professional responsibilities with household and caregiving roles became particularly challenging, often disrupting their personal lives and overall well-being.

“*Not every day, but on some days, I feel like crying because of the workload. I have three kids, and all of them are going to school. I need to take care of them before going to the field.” ~ ASHA 2*

Theme 4: motivation and support factors

Despite multiple challenges, participants remained strongly motivated by their commitment to community welfare. Many participants expressed a deep sense of responsibility toward the women and families they served, and often viewed their work as a form of social responsibility rather than just employment. The subthemes are given below.

Commitment to Community Welfare

Frontline health workers expressed a strong sense of social commitment toward the well-being of their own community. Many expressed their role as a responsibility to support needy women and their families during pregnancy and childbirth. This sense of social duty motivated them to continue working despite of these difficulties.

“*It is also my responsibility to improve the well-being of pregnant women. You may have heard a lot of news about infant deaths here, so they really need support. The workload is high, but at the same time, I take pride in my work.” ~ ASHA 5*

Personal Satisfaction From Positive Maternal Outcomes

Participants reported that witnessing positive maternal outcomes, such as safe deliveries, improved maternal health, and healthy newborns, brought them deep personal and professional satisfaction. These successes helped them feel that their efforts were meaningful and encouraged them to persist in challenging working conditions.

“Every day I feel that I have done something good for others. With my limited social conditions, I did my best for my people.” ~ ASHA 4

Social Recognition Within the Community

Acceptance, recognition, and respect from society were identified as strong motivating factors. Being deeply trusted and consulted by families strengthened workers’ satisfaction, confidence, and reinforced their sense of purpose. This social acceptance also helped build stronger relationships, making health interventions more effective in the indigenous reproductive health.

“My husband is very supportive. Not only me even he gets a lot of respect from the people. On some of his holidays, he comes with me to help. He ride the bike, so everyone knows him. Even after years, people still ask him about me.” ~ ASHA 5

Support from Peers and Informal Networks

Support and encouragement from other ASHA and AWWs, supervisors, and local health staff played a crucial role in coping with work-related stress. Every month, they had panchayat- or block-level meetings. These meetings provided an opportunity for information sharing and ventilation with each other. Informal conversations, shared experiences, and mutual encouragement helped workers manage emotional challenges and reduce feelings of isolation in remote tribal areas.

*“We are a team. Once or twice a month, we meet up. It is a great opportunity to share our problems. All the ICDS supervisors and *Local Self Government Department (*LSGD) members are very supportive because they know our struggles.” ~ AWW 5*

## Discussion

This study highlights an in-depth understanding of the experiences of frontline health workers delivering maternal and reproductive health services in a tribal setting in Kerala. The findings show that frontline health workers function far beyond their formally defined roles, acting as educators, mediators, counsellors, problem-solvers, and emotional supporters within complex socio-cultural and geographical contexts.

The study highlights that frontline health workers are central functionaries of community-level maternal and reproductive health services in limited-resource settings, particularly in tribal regions. Consistent with previous research, their roles extend well beyond clinical or task-oriented responsibilities and include sustained community engagement, advocacy, and follow-up care [28,29]. This study found that home visits emerged as a major strategy for health service delivery, particularly in settings where institutional care is underutilized due to distance, cost, fear, or cultural hesitation. Similar findings have been reported in various studies from other low- and middle-income countries where community-based outreach by frontline workers has been shown to improve positive perinatal outcomes such as early pregnancy registration, antenatal care utilization, and continuity of care [30,31]. Due to various cultural and family dynamics in tribal families, health-related decisions are rarely made by women alone. By considering the family as a unit, frontline workers help negotiate social norms and improve acceptance of medical care, thereby strengthening maternal health outcomes [32]. The holistic approach adopted by these workers engaging pregnant women’s family members reflects an important contextual adaptation

This study emphasizes cultural competence as a core skill in grassroots health work. Frontline workers shared a similar cultural background, language, and lived familiarity with tribal customs, which enabled them to navigate beliefs about pregnancy, childbirth, and illness. Rather than challenging traditional practices, workers developed a negotiated approach that balanced respect for tribal cultural beliefs with the promotion of safer health behaviors. The existing literature highlights that culturally insensitive interventions and healthcare approaches may increase resistance and distrust among indigenous populations [30].

The findings also show that traditional explanations for pregnancy complications, or maternal mental health risks such as beliefs related to the evil eye, black magic, or spirit possession, continue to influence health-seeking behaviour. Such perceptions often lead to delays in antenatal check-ups and increase the risk of adverse maternal and neonatal outcomes. Frontline workers’ skills of dealing with these beliefs rather than challenging them appear important in facilitating gradual change. This emphasises the importance of experiential and relational knowledge, which is essential for effective community health service delivery in tribal settings [33].

Previous researchers found that the geographical isolation and transportation barriers significantly impact health service delivery in tribal and remote health settings [34,35]. Similarly, the present study also emphasizes that the difficult terrain, seasonal inaccessibility, and exposure to wildlife added more physical risk and emotional strain to the delivery of routine services. These challenges not only delayed antenatal visits but also increased anxiety and fatigue among workers. Despite these challenges, frontline health workers adapted problem-solving strategies such as flexible scheduling, collaboration with self-help groups (Kudumbashree), and organizing outreach medical camps in remote hamlets. Poor infrastructure and resource gaps, especially during complicated medical emergencies and at night, further complicated their work. However, Attappady has received sustained policy and programmatic attention from the government system. The findings suggest that the availability of services alone does not guarantee accessibility or adequacy, especially in time-sensitive maternal health situations.

The study highlights the substantial work stress arising from staff shortages, wide geographical coverage, and the multiplicity of roles and responsibilities assigned to frontline workers. Beyond maternal and reproductive health, participants were actively involved in other community health initiatives, pandemic response, geriatric care, palliative care, data reporting, poverty alleviation programme, and community surveillance. Such overburden of work has been widely documented among ASHA and Anganwadi workers across India, with minimal remuneration and limited institutional recognition [36,37]. Repeated exposure to high-risk pregnancies, infant deaths, poverty, and one's own family distress, inadequate training for emotional coping and mental health support leads to stress, exhaustion, and symptoms of burnout in frontline workers. As most frontline health workers are women, the dual responsibility of professional work and domestic caregiving increases their stress and strain.

Several policies and programmes have been initiated by the government through local self-governing agencies and Primary Health Centres to address issues related to cultural competence and improve healthcare delivery in tribal settings. Frontline health workers receive regular training and are supervised by medical professionals as part of these efforts. However, these approaches largely rely on standardized models of communication and service delivery, with limited attention to the complex socio-cultural realities of indigenous communities. As a result, culturally adapted training and treatment modules remain inadequate, limiting the effectiveness of these interventions in practice [38]. At the same time, the government has made substantial investments in strengthening healthcare infrastructure through initiatives such as the National Health Mission and Ayushman Bharat, leading to the upgrading of Primary Health Centres into Ayushman Mandirs with improved facilities and modern medical equipment [39,40]. There has also been a strong emphasis on expanding access to healthcare through insurance schemes aimed at reducing financial barriers. Despite these significant efforts, multiple challenges continue to persist at the grassroots level [41]. Frontline health workers still face staff shortages, excessive workload, inadequate and irregular remuneration, and limited supportive supervision, which affect both their performance and well-being. These issues highlight a critical gap between policy-level initiatives and field-level realities, indicating that improvements in infrastructure and standardized training alone are insufficient without context-specific, culturally sensitive approaches and stronger organizational support systems.

Despite these challenges, frontline health workers show a remarkable resilience and sustained motivation in their profession. A strong sense of social responsibility, personal identification with the community, and the positive maternal outcomes served as powerful motivators. Social recognition and respect within the community reinforced their commitment, often extending to their families as well. Social acceptance appears to function as an informal but meaningful reward system compensating partially for systemic limitations. Studies have also found that strengthening peer-support networks and reflective spaces can serve as a low-cost yet effective strategy to enhance the capacity and well-being of frontline health workers and healthcare providers [42]. Similarly, the current study shows that peer support and informal relationships with colleagues are also crucial protective factors. Regular meetings, shared experiences, and collective problem-solving helped mitigate feelings of burnout and emotional distress among the service providers. This reflects that strengthening peer-support networks and reflective spaces could be a low-cost but effective way to enhance the ability and well-being of the service providers.

Limitations of the study

As a qualitative study conducted in a specific tribal setting in Kerala, the findings may have limited generalizability to other regions or populations with different socio-cultural and healthcare contexts. The findings relied on self-reported experiences of frontline health workers, which may be subject to recall bias and social desirability bias. In addition, the perspectives of other stakeholders, such as pregnant women, family members, or healthcare administrators, were not included, which could have provided a more comprehensive understanding of the issues. The small sample size (n = 18) and the single geographic setting further limit the transferability of the findings. Furthermore, methodological constraints such as limited transparency in the analytic process and the absence of triangulation may affect the robustness and credibility of the results. Constraints related to time and resources may also have limited the depth of exploration of certain emerging themes.

Therefore, future studies should focus on incorporating multiple stakeholder perspectives, ensuring greater transparency in data analysis, employing triangulation techniques, and using mixed-method approaches to enhance the comprehensiveness and generalizability of findings related to maternal and reproductive health service delivery in tribal settings.

## Conclusions

Overall, this study emphasizes that frontline health workers are not just service providers but key agents of social change within tribal maternal and reproductive health systems. Their culturally grounded practices, resilience, and commitment help to reduce the gaps between health systems and tribal communities. Recognizing, valuing, and supporting their work is essential for advancing equitable maternal health outcomes and reducing persistent health inequities among tribal populations.

This study highlights that there is a strong need to formally acknowledge and support the services of frontline health workers, particularly in tribal and resource-limited settings. Second, training programs should move beyond technical skills to include cultural competence, communication strategies, and one's own self-care. Addressing workload problems through adequate staffing, realistic demands, and supportive supervision is crucial to preventing burnout and work stress. Finally, strengthening the health infrastructure and emergency referral systems in tribal areas remains important for the elimination of preventable maternal and neonatal risks.

## Figures and Tables

**Figure 3:**
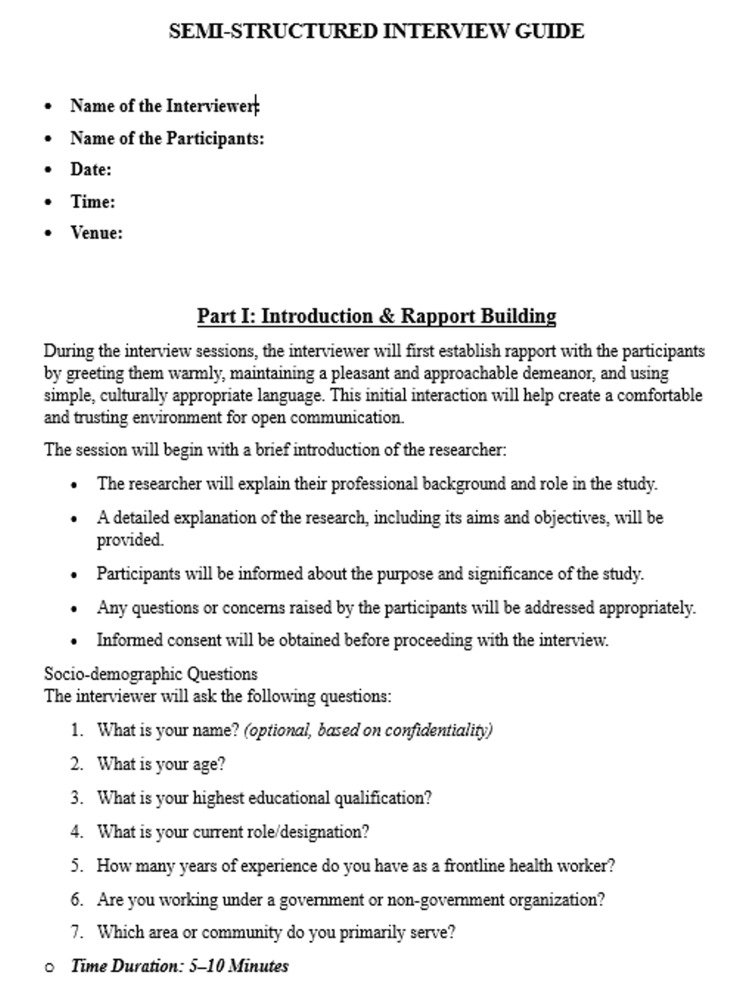
Semi-structured interview guide (Page 1)

**Figure 4:**
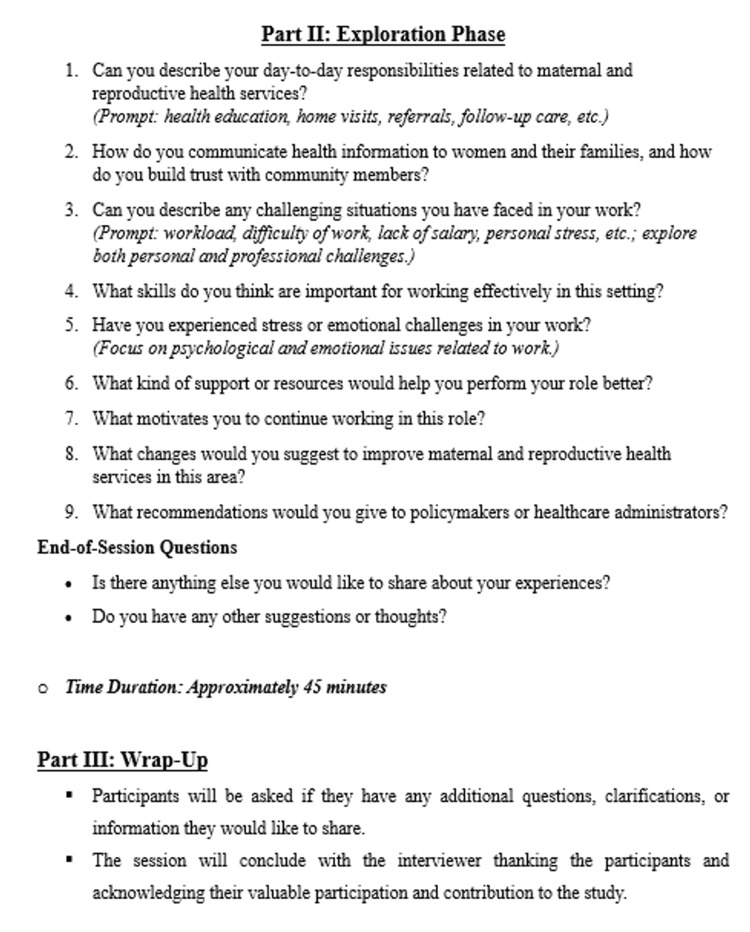
Semi-structured interview guide (Page 2)

**Figure 5:**
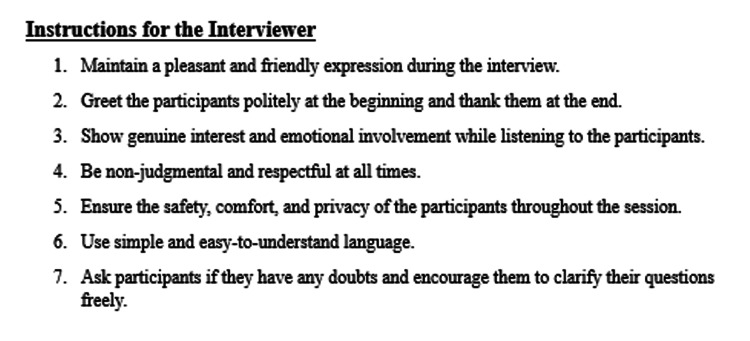
Semi-structured interview guide (Page 3)
